# A Service Evaluation of the Operative Length of Anterior Cruciate Ligament Reconstruction and Associated Procedures

**DOI:** 10.7759/cureus.81278

**Published:** 2025-03-27

**Authors:** Tomos Mather, Luka Jovanovic, Florence Bradshaw, Harshvir Grewal, Benjamin D Gompels, Simone Castagno, Tim Baker, Joel Melton, Arman Memarzadeh, Stephen McDonnell

**Affiliations:** 1 Division of Trauma and Orthopaedics, School of Clinical Medicine, University of Cambridge, Cambridge, GBR; 2 Division of Trauma and Orthopaedics, Lister Hospital, East and North Hertfordshire NHS Trust, Stevenage, GBR; 3 Department of Surgery, University of Cambridge, Cambridge, GBR; 4 Division of Trauma and Orthopaedics, Addenbrooke’s Hospital, Cambridge University Hospitals NHS Foundation Trust, Cambridge, GBR; 5 Division of Trauma and Orthopaedics, Cambridge University Hospitals NHS Foundation Trust, Cambridge, GBR

**Keywords:** acl reconstruction, girft (getting it right first time), lateral extra-articular tenodesis (let), operative time, patient outcomes

## Abstract

Background

The Getting It Right First Time (GIRFT) initiative emphasises pathway efficiency to achieve optimal surgical outcomes. While anterior cruciate ligament (ACL) reconstruction is common, the factors influencing operative duration and their relationship with patient characteristics and outcomes remain poorly understood, especially concerning complex procedures such as lateral extra-articular tenodesis (LET).

Methodology

This service evaluation analysed 157 ACL reconstructions at a major UK teaching hospital from 2019 to 2023. Procedures were categorised as isolated ACL reconstruction, ACL with meniscal repair, ACL with LET, or ACL and combined procedures. A multiple regression analysis evaluated predictors of operative time while assessing perioperative outcomes and readmission rates.

Results

Age independently predicted shorter operative times (-0.696 minutes/year, p = 0.005). LET procedures exhibited longer operative times but were mainly performed on younger, lower body mass index patients. An American Society of Anesthesiologists III status had an impact on the length of stay (hazard ratio (HR) = 0.440, 95% confidence interval (CI) = 0.276-0.704, p = 0.001), and ACL + LET procedures (HR = 0.466, 95% CI = 0.250-0.866, p = 0.016) were associated with significantly prolonged stays. Postoperative complications occurred in 6.4% (10/157) of cases, with significant age-related patterns observed. Affected patients were notably older (mean = 46.0 years, 95% CI = 39.2-52.8) compared to those without complications (mean = 26.6 years, 95% CI = 24.9-28.3).

Conclusions

This GIRFT-aligned analysis shows that age and procedure type significantly affect the duration of ACL reconstruction, highlighting distinct patterns in LET procedures. These findings indicate opportunities for optimising pathways through scheduling and procedure-specific postoperative protocols, particularly for complex cases.

## Introduction

The anterior cruciate ligament (ACL) is critical in maintaining knee stability and function, particularly during dynamic movements involving pivoting, twisting, or sudden direction changes [1]. ACL injuries are prevalent, especially among young athletes, and often lead to significant functional impairment [1-3]. Considering the critical role of the ACL in knee biomechanics, accurate and effective reconstruction after an ACL rupture is essential to restore normal knee function and prevent long-term complications such as joint degeneration and instability [2,4,5].

Despite the frequency of ACL reconstruction (ACLR) and its generally favourable outcomes [6-8], the procedure carries significant postoperative risks. Major independent risk factors for ACLR complications include ethnicity, the use of anaesthesia, and a history of bleeding disorders; however, the duration of the surgery itself is a critical factor that is often overlooked [9,10]. Prolonged operation times have been associated with an increased risk of complications, including deep vein thrombosis, surgical site infections, sepsis, extended hospital stays, and higher rates of re-admissions [10,11]. Therefore, assessing the duration of ACL and concomitant procedures while identifying the factors that influence these durations is crucial for improving the ACL repair pathways and outcomes within the NHS.

Getting It Right First Time (GIRFT) is an initiative to improve NHS surgical outcomes by evaluating operation times, patient pathways, and hospital processes. The GIRFT guidelines emphasise optimising surgical pathways and minimising operative times to enhance patient outcomes and streamline hospital operations. This service investigation seeks to evaluate the operative duration and associated outcomes of various ACL repair procedures in line with the GIRFT recommendations [12].

This service evaluation aims to analyse the intraoperative duration of ACLR, understand the factors influencing the length of operation times, and evaluate their impact on patient outcomes. The goal is to provide evidence-based recommendations for optimising ACLR pathways in alignment with GIRFT guidelines.

## Materials and methods

Study design and setting

We conducted a retrospective service evaluation of ACLRs at a tertiary teaching hospital between April 2019 and December 2023, focusing on GIRFT quality metrics. The primary outcome measures were day-case rate, operation time, length of stay, reoperation rates, and waiting times. The evaluation was registered locally (PRN12527) and handled according to local data protection regulations.

Study participants

Clinical coding datasets identified eligible patients. Five consultant orthopaedic surgeons performed all procedures. The exclusion criteria included incomplete surgical documentation (missing procedure codes and timing data), insufficient demographic information, unresolvable data inconsistencies, and emergency procedures. Data inconsistencies were resolved through a manual review of electronic hospital records.

Procedures were categorised into the following four distinct groups: ACL only (isolated ACLR), ACL with meniscus (ACLR with meniscal repair, including medial, lateral, or both menisci), ACL with lateral extra-articular tenodesis (LET) (ACLR with LET), and ACL with both meniscus and LET (ACLR with both meniscal repair and LET). Complex procedures were defined as ACLRs involving additional procedures such as meniscal repair and/or LET.

GIRFT guidelines

Key GIRFT metrics included the day-case rate, which refers to the proportion of patients discharged on the same calendar day; length of stay, measuring the time from admission to discharge; and operation time, defined as the duration from the first incision to closure. Additional metrics encompassed the re-admission rate, capturing unplanned returns within six months, and the reoperation rate, which measures further knee surgeries within six months.

Data collection and pre-processing

Data integration was performed across four core files for primary ACLRs, including coded procedure data, timing data that captured operation duration and theatre utilisation metrics, performance metrics encompassing clinical outcomes and GIRFT indicators, and demographic information, including patient characteristics and risk factors.

Missing values were handled according to their type. Surgery-specific identifiers (LogID) ensured accurate linkage between procedure, timing, and performance datasets, while hospital Medical Record Numbers (MRNs) achieved patient-level linkage.

Quality controls included data cleaning, where date and time formats were standardised to accurately calculate the length of stay and operation duration. Outlier detection was conducted by flagging extreme values, such as body mass index (BMI) greater than 50 kg/m^2^ or operation durations more than three standard deviations from the mean, for manual review.

Statistical analysis

Statistical analyses were performed using Python 3.13.1. Initial distribution analysis using Shapiro-Wilk tests revealed significant non-normality for all key variables (age: w = 0.940, p < 0.001; BMI: w = 0.977, p = 0.018; operation time: w = 0.983, p = 0.049; length of stay: w = 0.830, p < 0.001), informing the choice between parametric and non-parametric approaches. BMI non-normality was further confirmed using D’Agostino’s test (x^2^ = 8.95, p = 0.011) and Anderson-Darling test. Length of stay non-normality was further confirmed using D’Agostino’s test (x^2^ = 56.8, p < 0.001) and Anderson-Darling test. Further information on the statistical methods utilised can be found in the Appendix.

One-way analysis of variance was used for categorical comparisons where assumptions were met, revealing significant effects of procedure type on operation time (F = 12.0, p < 0.001), particularly for LET procedures (F = 28.3, p < 0.001) and meniscal repairs (F = 9.8, p = 0.002). Effect sizes and 95% confidence intervals (CIs) were reported for all significant comparisons.

Key relationships analysed (p < 0.05) included operation time versus length of stay (r = 0.16, p = 0.041), age versus operation time (r = −0.31, p < 0.001), and BMI versus operation time (r = −0.17, p = 0.046). Sensitivity analyses included assessment of outliers and handling of missing data. Detailed statistical methods and software specifications are provided in the Appendix.

Ethical considerations

This project was registered as a service evaluation (PRN12527), and all patient data were fully de-identified before analysis in accordance with local data protection regulations.

## Results

Cohort characteristics and procedure distribution

**Figure 1:**
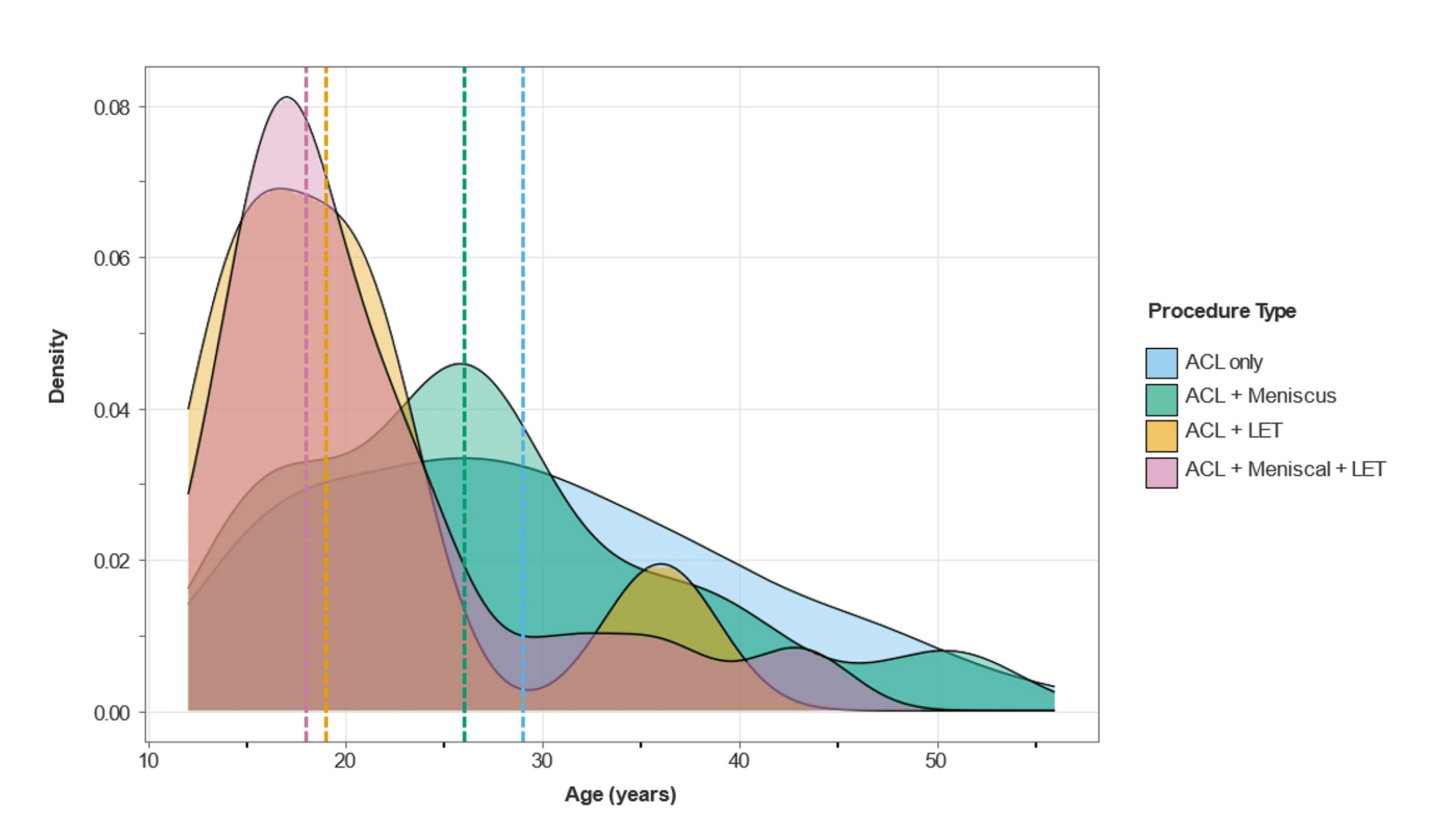
Age distribution of 157 ACL reconstruction patients by procedure type. The distribution shows a positive skew (0.71), reflecting younger patient predominance. ACL = anterior cruciate ligament; LET = lateral extra-articular tenodesis

A total of 157 patients underwent primary ACLR between April 2019 and December 2023. The cohort demonstrated distinct age-related patterns in surgical complexity, with a mean age of 27.0 years (SD = 10.3 years, range = 12-56). As shown in Figure 1, younger patients were more likely to undergo complex procedures, particularly those involving LET. ACL-only procedures (n = 73, 46.5%) involved older patients (mean = 29.2 years, SD = 10.6 years), while ACL and combined procedures such as ACL + LET (n = 7, 4.5%) and ACL + meniscus + LET (n = 19, 12.1%) were predominantly performed on younger patients (mean = 20.3 years, SD = 7.6 years and mean = 21.1 years, SD = 7.9 years, respectively). ACL + meniscus procedures (n = 58, 36.9%) exhibited intermediate age characteristics (mean = 27.1 years, SD = 10.1 years). The patient demographics are summarised in Table 1.

**Table 1:** Patient demographics according to different procedure type and ASA classification. Please be aware the ASA percentages for each procedure may not add up to 100%, which is due to ASA not being recorded within the patient notes. ACL = anterior cruciate ligament; LET = lateral extra-articular tenodesis; ASA = American Society of Anesthesiologists; IQR = interquartile range

Procedure type	N (%)	Mean age (SD)	Age range	Median BMI (IQR)	ASA I (%)	ASA II (%)	ASA III (%)
ACL only	73 (46.5%)	29.2 (10.6)	13–56	26.1 (24.5-29.8)	56.16	36.99	1.37
ACL + meniscus	58 (36.9%)	27.1 (10.1)	13–52	25.8 (22.2-28.6)	65.52	32.76	0.00
ACL + LET	7 (4.5%)	20.3 (7.6)	14–36	22.5 (21.0-25.1)	57.14	42.86	0.00
ACL + meniscus + LET	19 (12.1%)	21.1 (7.9)	12–43	24.7 (21.5-29.7)	73.68	21.05	0.00

**Figure 2:**
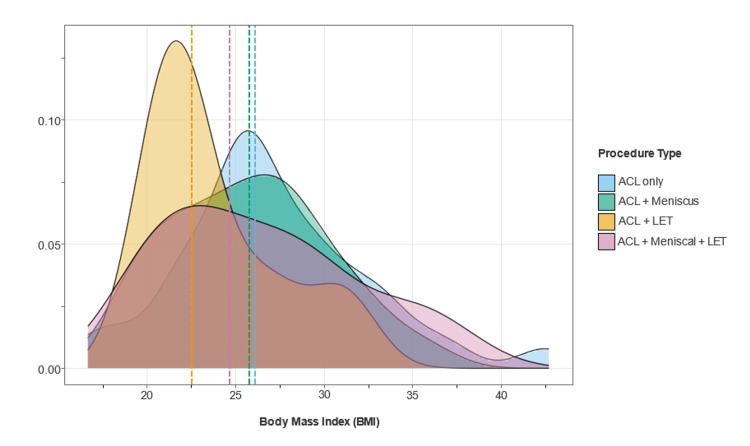
BMI distribution across procedure categories. Analysis of 157 patients shows BMI ranging from 16.6 to 42.7 kg/m^2^ with a mean of 26.4 kg/m^2^. Distribution spans underweight to obese categories with moderate positive skew and slight kurtosis. No significant procedure-specific patterns were observed, suggesting the minimal influence of BMI on surgical approach selection. BMI = body mass index; ACL = anterior cruciate ligament; LET = lateral extra-articular tenodesis

As illustrated in Figure 2, BMI distribution showed significant deviation from normality (Shapiro-Wilk w = 0.977, p = 0.018; D’Agostino x^2^ = 8.95, p = 0.011) with no procedure-specific patterns (Kruskal-Wallis, p > 0.05), suggesting body habitus did not influence surgical approach selection.

Operative time and surgical complexity

**Figure 3:**
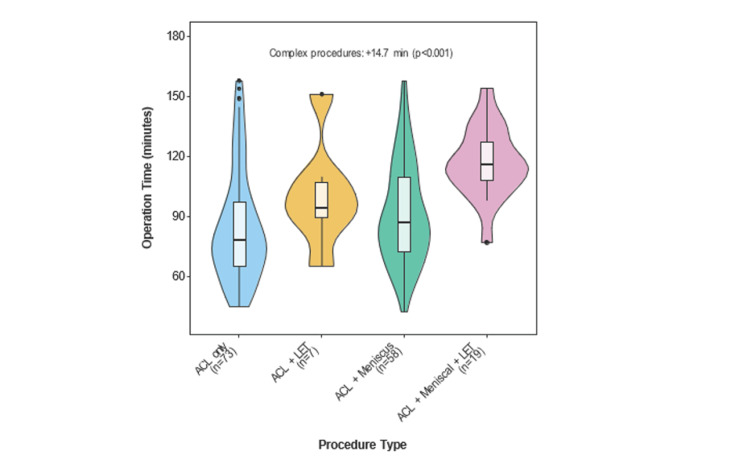
Operation time distribution by procedure type. Analysis of 157 procedures shows significant variation in duration across groups. ACL = anterior cruciate ligament; LET = lateral extra-articular tenodesis

Operation time was measured from knife to skin to closure and increased with procedural complexity (Figure 3). ACL-only procedures were completed in a median time of 78 minutes, whereas the addition of meniscal repair increased the duration to 87 minutes. Incorporating LET further extended operation times, with ACL + LET procedures requiring 94 minutes and ACL + meniscus + LET procedures taking 117 minutes. These differences were statistically significant (Kruskal-Wallis, p < 0.001), with post-hoc analysis revealing significant differences between all groups (p < 0.001) except between ACL + LET and ACL + meniscus procedures (p = 0.067). Table 2 shows a summary of the operative duration based on the procedure.

**Table 2:** Operative duration based on procedure and time increase against an ACL baseline. ACL = anterior cruciate ligament; LET = lateral extra-articular tenodesis; IQR = interquartile range

Procedure type	Median time/minute (IQR)	Time increase (vs ACL)
ACL only	78.0 (32.0)	Reference
ACL + meniscus	86.5 (37.5)	+8.5 minutes
ACL + LET	94.0 (17.5)	+16.0 minutes
ACL + meniscus + LET	117.0 (26.5)	+39.0 minutes

**Figure 4:**
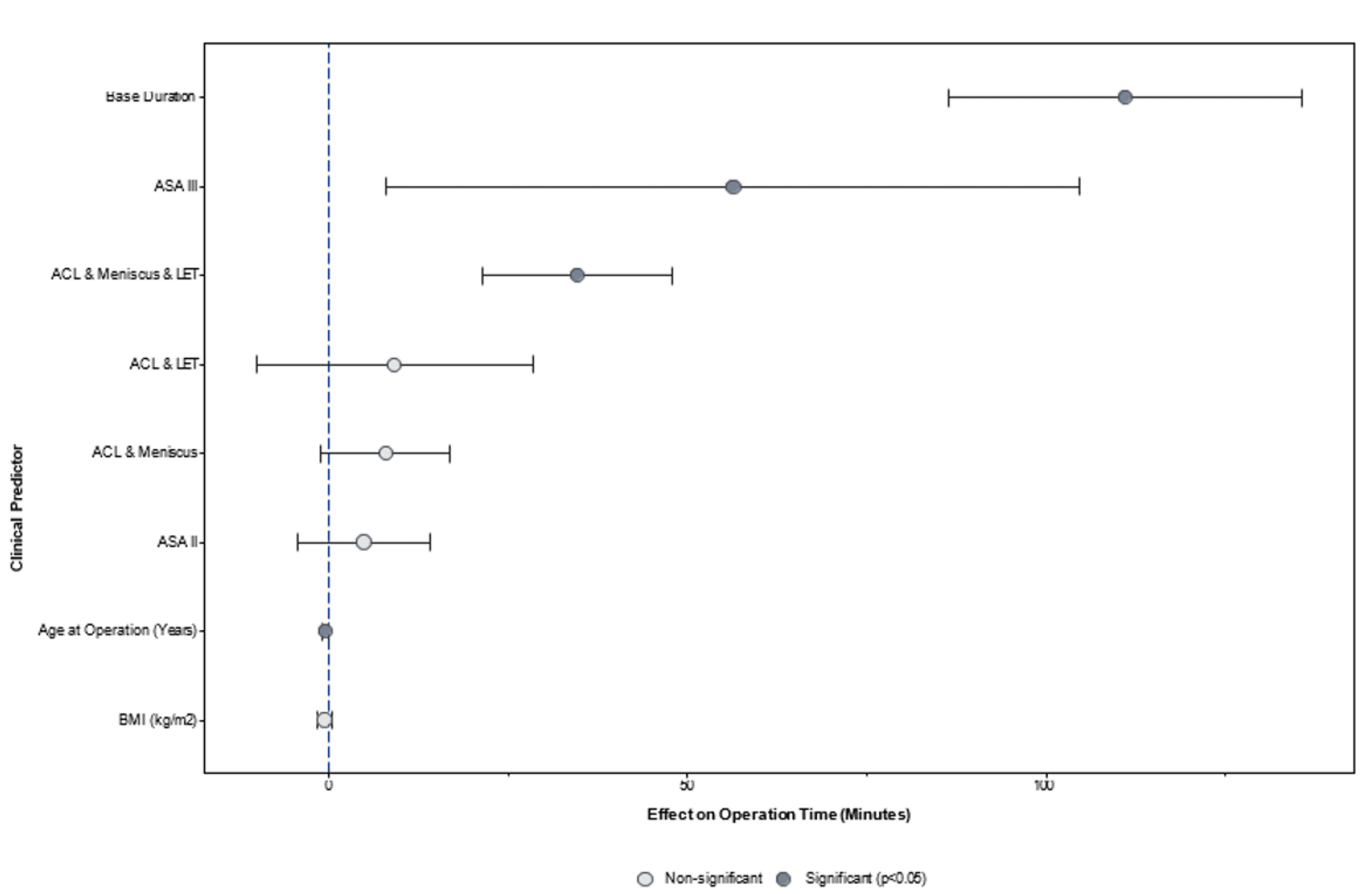
Forest plot of predictors influencing operation time. Key predictors include ASA III status and ACL + meniscus + LET procedures. ACL = anterior cruciate ligament; LET = lateral extra-articular tenodesis; BMI = body mass index; ASA = American Society of Anesthesiologists

**Figure 5:**
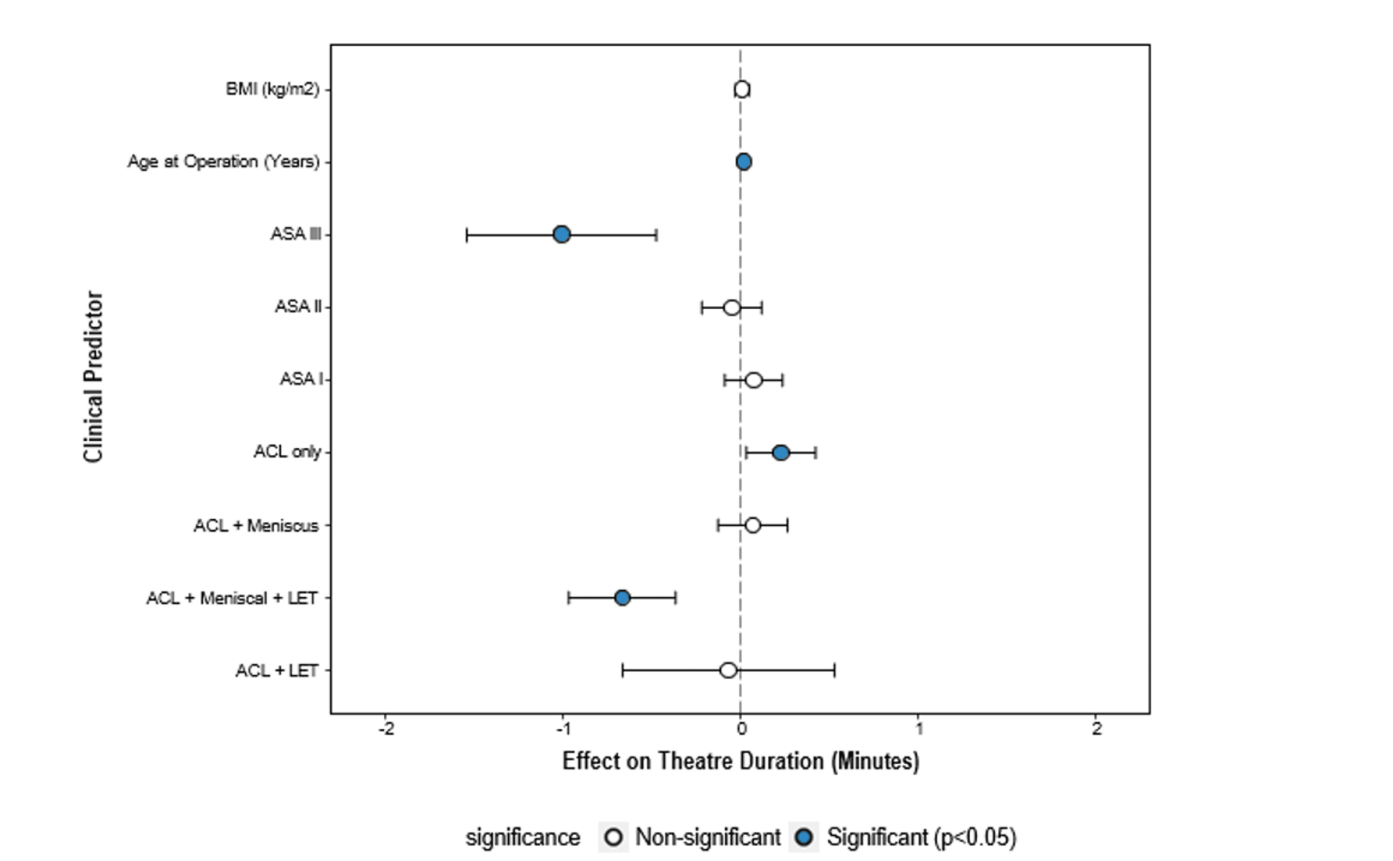
Forest plot showing adjusted effects on operative time, controlling for patient and procedural factors. Age demonstrates a consistent negative correlation with operation time. ACL = anterior cruciate ligament; LET = lateral extra-articular tenodesis; BMI = body mass index; ASA = American Society of Anesthesiologists

As presented in Figure 4, multivariate analysis demonstrated that younger age consistently correlated with longer operation times (r = −0.31, p < 0.001), likely reflecting a higher proportion of additional procedures in this demographic, such as undergoing an LET. Complex procedures added an average of 14.7 minutes to operation time (95% CI = 5.6-23.8, p < 0.001), independent of other factors. After controlling for patient characteristics (Figure 5), these patterns remained significant, indicating that surgical complexity is the primary determinant of theatre resource utilisation.

Length of stay

**Figure 6:**
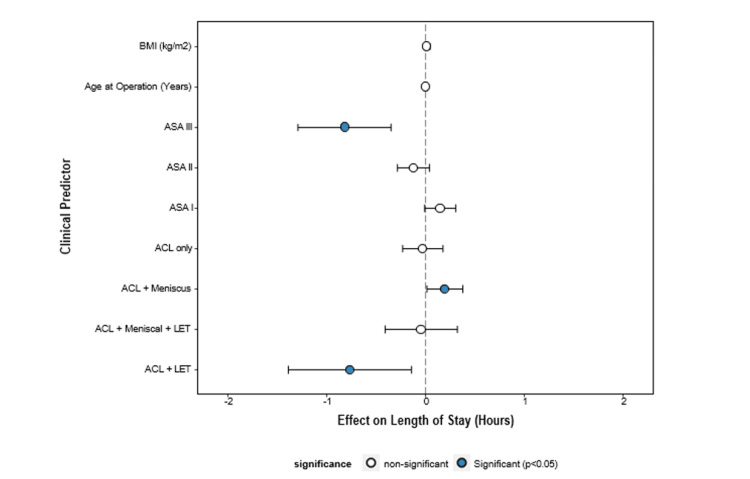
Cox proportional hazards analysis of length of stay predictors. Forest plot demonstrating factors influencing hospital stay duration. ASA III status and ACL + LET procedures were associated with extended inpatient stays. ACL = anterior cruciate ligament; LET = lateral extra-articular tenodesis; BMI = body mass index; ASA = American Society of Anesthesiologists

An American Society of Anesthesiologists (ASA) III status influenced the length of stay (hazard ratio (HR) = 0.440, 95% CI = 0.276-0.704, p = 0.001), and ACL + LET procedures (HR = 0.466, 95% CI = 0.250-0.866, p = 0.016) were associated with longer stays (Figure 6).

Patient or operation factors were not the only factors affecting the length of stay. There was some relationship between the day of surgery and the length of stay. Friday procedures (n = 47, mean = 25.0 hours, SD = 15.2 hours) demonstrated significantly longer stays compared to other weekdays (mean = 19.4 hours, SD = 11.4 hours). Table 3 summarises various predictors influencing the length of stay.

**Table 3:** Length of stay analysis with predictors and significance. ACL = anterior cruciate ligament; LET = lateral extra-articular tenodesis; BMI = body mass index; ASA = American Society of Anesthesiologists; CI = confidence interval

Factor	Effect on length of stay (hours)	95% CI	Significance (p < 0.05)
ASA III	+18.27	-5.24 to 41.79	Non-significant
Base duration	+13.29	-1.75 to 28.33	Non-significant
ACL + LET	+9.65	-0.47 to 19.77	Non-significant
October	+8.10	2.43 to 13.78	Significant
Friday	+5.59	0.84 to 10.33	Significant
ASA II	+3.99	-0.48 to 8.47	Non-significant
September	+2.76	-2.42 to 7.93	Non-significant
March	+2.05	-6.12 to 10.22	Non-significant
July	+1.34	-5.91 to 8.59	Non-significant
February	+0.81	-7.74 to 9.35	Non-significant
Thursday	+0.69	-5.69 to 7.07	Non-significant
May	+0.29	-7.97 to 8.55	Non-significant
Month number	+0.27	-0.68 to 1.21	Non-significant
Tuesday	+0.13	-10.08 to 10.34	Non-significant
Age at operation (years)	+0.04	-0.17 to 0.25	Non-significant
BMI (kg/m^2^)	+0.04	-0.43 to 0.51	Non-significant
ACL + meniscus + LET	-1.26	-7.78 to 5.25	Non-significant
ACL + meniscus	-1.81	-6.19 to 2.57	Non-significant
November	-1.79	-8.04 to 4.47	Non-significant
August	-1.83	-7.43 to 3.77	Non-significant
April	-2.28	-13.86 to 9.30	Non-significant
June	-2.72	-10.58 to 5.14	Non-significant
Wednesday	-3.47	-9.37 to 2.44	Non-significant
December	-6.45	-13.09 to 0.19	Non-significant

Complications and outcomes

Postoperative complications occurred in 10/157 (6.4%) of cases, with significant age-related patterns observed (Table 4). Affected patients were notably older (mean = 46.0 years, 95% CI = 39.2-52.8) compared to those without complications (mean = 26.6 years, 95% CI = 24.9-28.3). This age difference of 19.4 years was statistically significant (p = 0.010).

**Table 4:** Postoperative complication rate and classification. ACL = anterior cruciate ligament; CI = confidence interval

Factor	Complication rate, N (%)
Overall rate	10 (6.4%)
ACL only	3 (4.1%)
Complex procedures	7 (8.3%)
Hemarthrosis	4 (2.5%)
Infection	3 (1.9%)
Mean age (with complications)	46.0 (95% CI = 39.2–52.8)
Mean age (without complications)	26.6 (95% CI = 24.9–28.3)

Complication rates varied by procedure complexity. Complex reconstructions (ACL + meniscus ± LET) showed higher complication rates (n = 7, 8.3%) compared to isolated ACL procedures (n = 3, 4.1%, p = 0.038). The most common complications were hemarthrosis (n = 4, 2.5%) and infection (n = 3, 1.9%). All complications were successfully managed without the need for revision surgery.

## Discussion

Demographics of patients receiving ACLR

The demographic characteristics of patients undergoing ACLR were notably uniform, showing minimal variation in age, BMI, and ASA status across groups. This homogeneity enhances the reliability of comparative analyses. The generally good preoperative health likely contributed to the low rates of complications and re-admissions.

BMI distribution and its implications for procedure selection

The analysis of BMI distribution (Figure 2) highlights the trend of patient BMI concerning the type of ACL procedure conducted. A higher percentage of normal-weight and overweight patients underwent ACL + LET procedures compared to other methods, whereas only 14.0% of obese patients opted for ACL + meniscal repairs. Conversely, ACL-only procedures demonstrated a more uniform BMI distribution. This difference may indicate specific patient selection criteria or surgical preferences. For example, LET is often recommended for active individuals with lower BMI, particularly those experiencing multiple ligament injuries, elevated activity levels, or prior unsuccessful reconstructions [13]. It is also suggested for patients displaying signs of anterolateral rotatory instability, especially if they have a positive pivot shift test or are athletes prone to reinjury [14,15]. Consequently, LET procedures are performed more frequently in individuals with lower BMIs who are likely to meet the indication criteria. While BMI did not significantly affect procedure duration, this tendency for lower BMI patients to receive LET could indirectly impact operative times and outcomes, highlighting its possible influence on surgical planning, including anticipated procedure durations and perioperative care considerations.

ASA

Although the coefficient for ASA II (9.0 minutes, p = 0.081) did not reach statistical significance, its magnitude indicates that patients with more severe systemic health issues may require substantially longer surgical times (Figures 4, 5). These findings highlight the importance of preoperative health status in surgical planning, as more complex cases may necessitate additional resources or adjustments in workflow. Future studies should involve a larger cohort of ASA III patients to investigate this relationship.

Age as a predictor for surgical times

Age demonstrated a statistically significant negative effect on procedure duration (p = 0.006), with each additional year associated with a 0.7-minute reduction in operative time (Figure 5). This finding suggests that younger patients may require longer surgeries due to additional concurrent procedures. Notably, age was the only significant predictor of procedure duration. The tendency for younger individuals to engage in high-impact sports [13,14] may explain the increased likelihood of multi-ligament injuries or the need for LET procedures, which can extend the operative time. This is supported by the reduced significance of age when comparing surgery types (Figure 3), implying that the longer operative times in younger patients are likely driven by the frequency of more complex procedures.

Interestingly, the ACL + LET procedures indicated a non-significant and weak negative correlation between age and operative time (r = −0.13, p = 0.714), suggesting that other procedural factors or patient characteristics may play a more substantial role in determining operative time. However, notable trends were evident in ACL-only and combined ACL, meniscus, and LET procedures. A statistically significant negative correlation (r = −0.26, p = 0.035) indicated shorter operative times associated with increasing age in ACL-only surgeries. Similarly, combined ACL, meniscus, and LET procedures displayed a moderate negative correlation (r = −0.36) with a slope of −1.0, although this did not achieve statistical significance. These shorter operative times for older patients likely reflect a decreased need for extensive surgical customisation or fewer additional interventions.

The rising frequency of LET procedures among younger patients may account for the overall trend; however, further research is necessary to clarify the effect of age on operative times. Examining surgical protocols across various age groups can improve pathway optimisation and efficiency according to GIRFT initiatives.

Observations on operation duration

Trends in operation duration (Figure 3) reveal the influence of procedural complexity. Surgical times are extended when an LET is included (Figure 3). However, unmeasured cofounders, such as surgeon experience or intraoperative challenges, may influence the operation duration but were not addressed in this study. Despite this, the observations made emphasise the necessity for careful planning when allocating operating theatre slots for such procedures. Recognising this trend can aid in optimising scheduling and minimising potential delays in surgical pathways.

Impact of procedure on length of stay

Despite procedural complexity and varying operation times, the length of hospital stay varied only slightly across ACLR procedures (Figure 6). These factors generally impact hospital stay durations, allowing for standardised discharge timelines. This finding supports streamlined patient management, reducing variability and facilitating efficient resource utilisation.

Non-elective re-admissions

The re-admission rates across all procedures were notably low, underscoring the efficacy of current ACLR treatment pathways in minimising postoperative complications. Specifically, the ACL + meniscus + LET combination demonstrated zero re-admissions in this cohort. However, slightly higher re-admission rates were observed in ACL/MM (n = 12, 20.7%) and ACL/LET (n = 1, 14.3%). These findings suggest that enhanced postoperative care or targeted follow-up may benefit patients undergoing these procedures.

It is crucial to contextualise these observations, as the addition of LET has been shown to reduce the risk of revision by 2.8 times in elite athletes after adjusting for sex and age [16]. Furthermore, LET has been associated with improved return to activity and better restoration of preoperative knee function compared to other surgeries [17]. Although the slightly higher re-admission rates observed in procedures involving LET may raise concerns, the technique offers substantial long-term benefits, including a reduced risk of revision, enhanced return to activity, and superior functional outcomes. These long-term advantages outweigh the short-term risks associated with the increased complexity of the procedure.

Optimising postoperative protocols and targeted follow-up for LET patients can mitigate early complications and maximise long-term benefits. Re-admission risk factors include age, female sex, and concurrent meniscal treatments. Identifying these at-risk groups enables tailored strategies to reduce complications and improve outcomes.

Further research is needed to assess whether operative times and complexity increase the re-admission risk. Studying patient activity levels could also offer valuable insights into enhancing postoperative strategies for high-risk individuals.

In-recovery time

Postoperative recovery times varied across procedures but did not directly correlate with operation duration or complexity (Figure 4). For instance, ACL with medial and lateral meniscus repair and LET had the shortest recovery time (55 minutes), whereas ACL with lateral meniscus repair had the longest (94 minutes). These findings highlight the potential for standardising recovery protocols, as factors beyond procedure type may be driving this variability. Identifying and addressing these factors could enhance patient satisfaction and efficiency in postoperative care.

GIRFT recommendations and procedural insights

LET Procedures

LET procedures present an area for pathway optimisation. Strategies to enhance patient outcomes include refining surgical techniques, re-evaluating the criteria for performing LET or improving postoperative support. Exploring these avenues could reduce re-admission rates and improve overall patient care. These pathways should also consider the influence of age and BMI on the likelihood of undergoing an LET procedure and assess whether this predictor can improve efficiency.

Standardisation of Recovery

The observed variation in recovery times underscores the potential for standardising postoperative protocols. As these discrepancies seem independent of procedural complexity, targeted postoperative analyses may uncover actionable areas to minimise variability, enhancing patient outcomes and procedural efficiency.

Low Admission Rates

The low re-admission rates observed across all procedures are a positive outcome of current ACLR pathways. Nevertheless, ongoing monitoring of factors influencing re-admission, particularly for complex surgeries involving LET, could further decrease these rates and enhance patient care.

Alignment With GIRFT Initiatives

This study aligns with the GIRFT initiative by identifying procedural trends and variability, thus highlighting opportunities for pathway optimisation. Specifically, the findings emphasise the importance of addressing variability in operation and recovery times, refining surgical techniques, and enhancing postoperative support, particularly for LET procedures. These efforts improve the efficiency and quality of ACLR pathways, benefiting patients and healthcare systems.

Gender Disaggregation Dataset

Within this dataset, no distinctions were made between genders due to the limited numbers and low statistical power if divided by gender. Given the substantially higher incidence of ACL injuries among females and the limited understanding of the potential anatomical variations and biomechanical, neuromuscular, and hormonal contributions [18], further assessment would be necessary to determine whether these conclusions are consistent across different genders. Future research may investigate whether additional complications, types of surgery, surgical times or functional outcomes differ in women based on these physiological differences.

## Conclusions

This service evaluation examined the factors influencing the durations of ACLR operations at a single centre. The duration of procedures, which encompasses both procedural and total theatre time, increased with LET, and younger patients typically underwent LET. This may explain the significant correlation between age and operation duration. Although GIRFT guidelines recommend scheduling five ACL procedures daily, this study suggests that incorporating additional procedures such as LET may require optimising the patient pathway or reducing the number of surgeries performed on that day.

## Appendices

Statistical analysis: Further information

The waiting time distributions were analysed using kernel density estimation with Gaussian kernels. Bandwidth selection adhered to Silverman’s rule of thumb, and reflection method boundary correction was applied to accommodate non-negative time constraints. The characteristics of the distribution were evaluated using the Kolmogorov-Smirnov test for normality assessment, the Anderson-Darling test for goodness of fit evaluation, and chi-square testing for comparisons of distributions across procedure types and time periods. GIRFT target compliance was assessed against the standard of 18 weeks (126 days).

Missing data were managed using a complete case approach, where records were preserved but excluded from specific analyses when the required data elements were unavailable. This conservative strategy was selected to uphold data integrity for GIRFT quality metrics reporting and ensure that findings were based solely on actual rather than imputed data. This method aligns with clinical audit requirements and prevents the introduction of potential bias in critical surgical metrics, which is vital given that missing surgical data often holds clinical significance.
